# IgG-NR2B—A Potentially Valuable Biomarker in the Management of Refractory Anti-NMDAR Encephalitis

**DOI:** 10.3390/ijms26020513

**Published:** 2025-01-09

**Authors:** Zuzana Števková, Georgi Krastev, Miroslav Mako, Zuzana Čierna

**Affiliations:** 1 Clinic of Neurology, Faculty Hospital Trnava, 917 02 Trnava, Slovakia; krastevg1@gmail.com (G.K.); miroslav.mako@fntt.sk (M.M.); 2 Clinic of Neurology in Trnava, Slovak Medical University, 833 03 Bratislava, Slovakia; 3 Faculty of Medicine, Comenius University in Bratislava, 813 72 Bratislava, Slovakia; 4 Jessenius Medical Faculty in Martin, Comenius University in Bratislava, 813 72 Bratislava, Slovakia; 5 Department of Pathology, Faculty of Health Care and Social Work, Trnava University and University Hospital, 917 02 Trnava, Slovakia; 6 Department of Pathology, Faculty of Medicine, Comenius University and University Hospital Bratislava, 811 08 Bratislava, Slovakia

**Keywords:** IgG-NR2B, anti-NMDAR encephalitis, biomarker

## Abstract

The autoantibodies against the NR1 subunit are well known in the pathomechanism of NMDAR encephalitis. The dysfunction of the NR2 subunit could be a critical factor in this neurological disorder due to its important role in the postsynaptic pathways that direct synaptic plasticity. We report a case of paraneoplastic anti-NMDAR encephalitis presented alongside very severe illness. Computed tomography (CT) of the brain, as well as FLAIR and T2-weighted MRI, was performed to rule out any other acute brain processes. A semi-quantitative method was applied to detect the presence of anti-NMDAR antibodies in the serum and CSF. A CT chest–abdomen–pelvis scan was performed that detected an ovarian teratoma. A histopathological examination was performed after a laparoscopic right-ovary cystectomy. Subsequent immunofluorescence immunohistochemical staining showed the expression of NMDA receptors of type NR2B. Treatment included first-line immunotherapy, second-line immunotherapy, tumor removal, and intrathecal injections with methotrexate and dexamethasone. The histological finding for our patient after tumor removal was ovarian teratoma. Hematoxylin–eosin (HE) staining revealed a characteristic spectrum of elements, including stratified squamous epithelium and fat tissue accompanied by neuroglial cells. Subsequent immunohistochemical staining showed an expression of NMDA receptors of type NR2B in different structures of the teratoma, including the neuroglial cells. The first-line immunotherapy following the tumor removal was insufficient in our patient. The paraneoplastic anti-NMDAR encephalitis with a coexpressed NR2B subunit on the neural cells of the ovarian teratoma may suggest a different inflammation process and could be the key factor in the pathomechanism and treatment of the refractory anti-NMDAR encephalitis.

## 1. Introduction

Anti-N-methyl-D-aspartate receptor encephalitis (anti-NMDARE) is an autoimmune disorder associated with ovarian teratoma in young women [1]. The main role in the pathogenesis of this neurological disorder is played by the N-methyl-D-aspartate receptors (NMDARs), especially the NR1 subunit, expressed by teratoma neuroglial tissue, which can induce an auto-immune response in the central nervous system [2,3]. However, some studies have reported cases without tumor findings, including sporadic teratomas without anti-NMDARE presentation [1]. Thus, available data suggest that the expression of NMDAR is not enough to induce anti-NMDARE [2]. The clinical presentation of NMDARE is characterized by a prodromal phase of a viral-like illness (fever, headache, respiratory and gastrointestinal symptoms) followed by psychiatric symptoms (memory loss, hyperreligiosity, agitation, psychosis, auditory and visual hallucinations), physical signs (dyskinetic movements, orofacial dyskinesias, epileptic seizures) and autonomic dysfunction [1,4]. The diagnosis of anti-NMDARE is established upon the detection of anti-NMDAR antibodies in serum and CSF. There are no established guidelines for its treatment, but first-line immunotherapy (corticosteroids, immunoglobulin (IVIG), plasma exchange (PLEX)), as in most other autoimmune inflammatory disorders, is recommended, including tumor removal [1,4]. Second-line immunotherapy (rituximab, cyclophosphamide, tocilizumab) may be needed in cases when the diagnosis of anti-NMDARE is delayed [4]. Intrathecal therapy with methotrexate may be a useful therapy in cases where there is an insufficient response to first- and second-line immunotherapy (Table 1) with poor functional outcomes [5]. Optimized treatment strategies have not yet been determined and randomized controlled trials are necessary to confirm the efficacy and safety of all forms of immunotherapy [6]. We report a case of paraneoplastic anti-NMDAR encephalitis that presented with very severe illness, where the patient did not respond to first- or second-line immunotherapy following tumor removal.

## 2. Case Report

A 25-year-old female with no prior serious issues in her medical or psychiatric history was admitted to a local infectious department for severe headache, nausea, and a fever (body temperature of 39.0 °C) which lasted for 4 days. Because of her headache, photophobia, phonophobia, and neck stiffness, acute meningitis was considered for the patient. We carried out computed tomography (CT) of the brain, as well as a brain magnetic resonance imaging (MRI) scan, with negative findings for any acute process. Initial laboratory studies, including a comprehensive metabolic panel and electroencephalogram (EEG), were unremarkable. After a few days, she began to develop psychiatric symptoms (she was confused, aggressive, and somnolent). She underwent a diagnostic lumbar puncture with a routine cerebrospinal fluid (CSF) examination (Table 2), with normal glucose findings (2.49 mmol/L) being noted, but she had an elevated protein expression (1.43 g/L) with monocytosis (723/µL).

Due to the patient’s psychiatric symptoms and the results of the routine biochemical CSF examination, herpes simplex virus (HSV) encephalitis was also considered, and Acyclovir (with a treatment dose of 5 g) was used as the first-line treatment option. However, a CSF polymerase chain reaction (PCR) assay was negative for HSV and also for other neurotropic viruses (Table 3).

**Figure 1:**
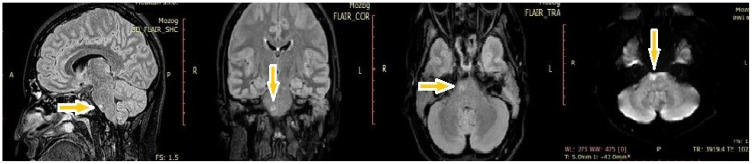
MRI findings of hyperintensities in the brainstem (yellow arrows) in FLAIR-weighted images and DWI.

After six days, the patient became increasingly somnolent and verbally unresponsive. A physical neurological examination revealed somnolence, orofacial dyskinesia, and spastic tetraplegia with generalized tonic–clonic seizure. She was transferred to the neurological intensive care unit (ICU) and underwent other examinations. Control brain MRI showed an abnormal focal brainstem lesion. FLAIR- and T2-weighted MRI showed that the lesion was pontine hyperintense and non-enhancing after the administration of gadolinium chelating agents. It also did not display restricted diffusion (Figure 1).

**Figure 2:**
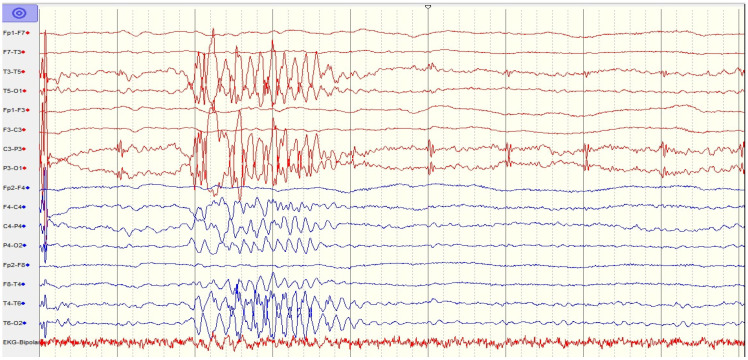
EEG findings of generalized PSW complexes.

Longer-range EEG monitoring (Figure 2) showed an epileptogenic pattern with generalized positive sharp waves (PSWs). However, after parenteral antiepileptic drugs (AEDs) were administered (levetiracetam, sodium valproate, and clobazam), the EEG results became worse, and supportive EEG findings confirmed convulsive status epilepticus. A semi-quantitative method was applied to detect the presence of anti-NMDAR antibodies in the serum and CSF, with a positive result (Table 4). After the diagnosis of anti-NMDAR encephalitis was confirmed, first-line immunotherapy including intravenous methylprednisolone (1 g daily for 5 days), two cycles of intravenous immunoglobulin (0.4 g/kg/day for 5 days during the first cycle and 2 g/kg/day for 3 days during the second cycle, with a distance of 6 weeks), and plasma exchange (one session every other day for two cycles) was administered, though this did not yield an adequate response. Using the Glasgow coma scale (GCS) to evaluate loss of consciousness, we found that she had a score of 4–5 with autonomic dysfunction (hypotension + tachycardia) and central hypoventilation. She was also noted to have brief episodes of tonic–clonic seizure activity necessitating critical anesthesiologist care. Acute respiratory failure led to emergent endotracheal intubation, including mechanical ventilation. Intravenous continuous anesthetic therapy with thiopental was administered for sedation, as well as for the treatment of refractory status epilepticus.

**Figure 3:**
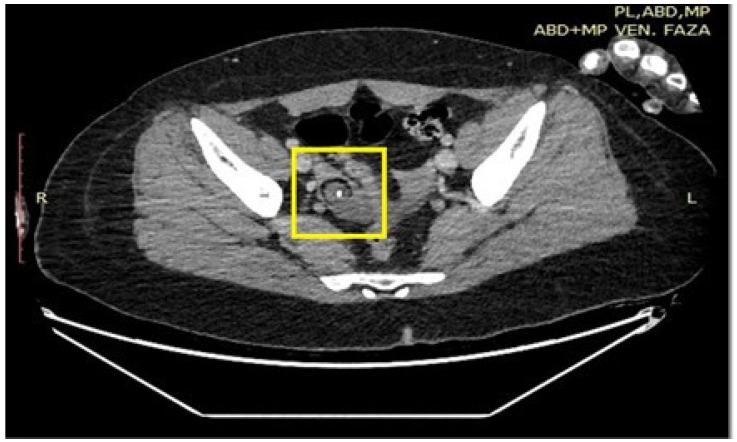
CT examination of the small pelvis, with a dermoid cyst (yellow box) found in the region of the right ovary.

A CT chest–abdomen–pelvis scan was requested for further evaluation, and in the right ovary a smaller-sized solid cystic mass-like lesion, more akin to a dermoid cyst, was found (Figure 3).

**Figure 4:**
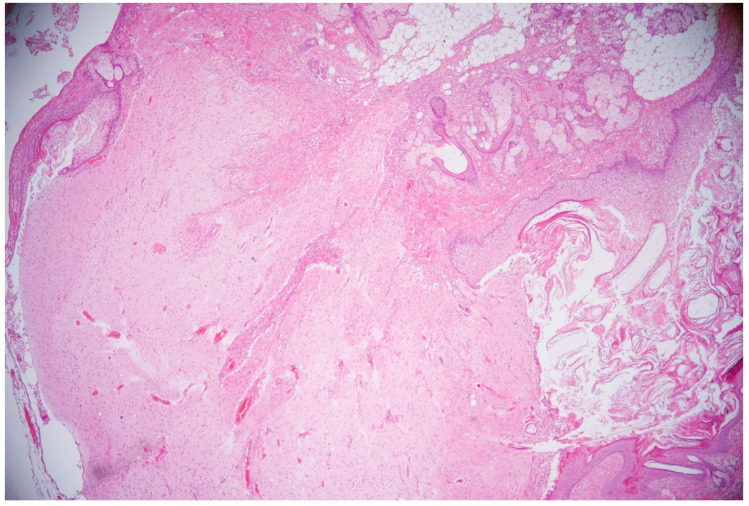
Histological appearance of mature ovarian teratoma composed of different structures—stratified squamous epithelium, cutaneous adnexal structures (sweat glands, pilosebaceous units with hair follicles and sebaceous glands), fat tissue, and neuroglial cells. HE, 40x magnification.

While there was no response to the first-line immunotherapy, second-line immunotherapy with rituximab (375 mg/m^2^/every two weeks for two cycles) was initiated, and this immunotherapy included the use of cyclophosphamide (after 2 months, a monthly dose of 750 mg/m^2^ was used). The patient also underwent a laparoscopic right-ovary cystectomy. After tumor removal, the histological findings revealed a mature ovarian teratoma. Hematoxylin–eosin (HE) staining revealed a characteristic spectrum of elements, including stratified squamous epithelium and cutaneous adnexal structures (sweat glands and pilosebaceous units with hair follicles and sebaceous glands) and fat tissue accompanied by neuroglial cells (Figure 4).

**Figure 5:**
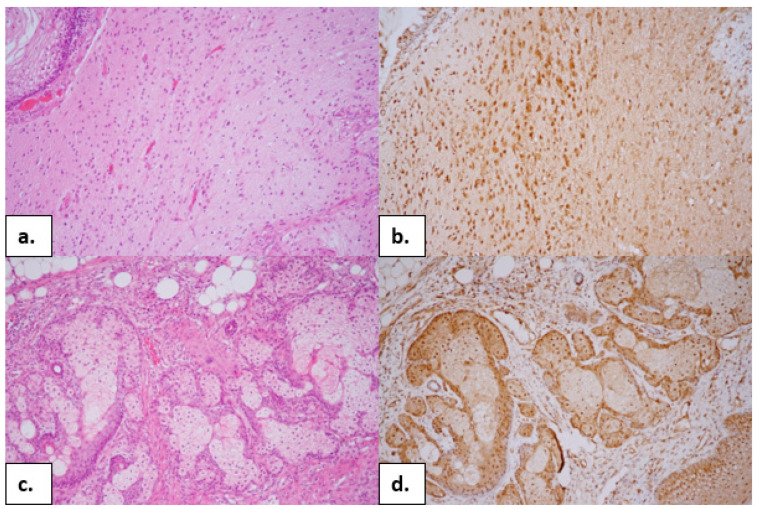
Detail of neuroglial cells ((**a.**), HE). Detail of sebaceous glands ((**c.**), HE). Immunohistochemical reaction of antibody against NMDA receptor type 2B, with diffused positivity (brown color) in neuroglial cells (**b.**) and sebaceous glands (**d.**); visualization DAB, 200x magnification.

Subsequent immunohistochemical staining showed an expression of the NR2B subunits of NMDARs in different structures of the teratoma, including the neuroglial cells (Figure 5).

No sustained improvement occurred after the initiation of either first- or second-line immunotherapies or after tumor removal (her GCS score was 3, her mRS score was 5). Our patient progressed into a state of unresponsiveness within 86 days. The control EEG recording showed a unique electrographic pattern with “extreme delta-brush”. There was an increased level of CSF anti-NMDAR antibodies with monocytosis (28). Due to excessive intrathecal antibody production with unsuccessful recovery from standard immunotherapy, we realized that these therapeutic strategies were insufficient in our patient. After the failure of the first- and second-line immunotherapies, an intrathecal application of methotrexate (15 mg weekly during the first two weeks, followed by a weekly dose of 10 mg), along with dexamethasone (20 mg), was carried out for 4 weeks. CSF and blood samples were collected before each intrathecal injection for the identification of anti-NMDAR antibodies (Table 4). After the second application of methotrexate (MTX) and dexamethasone (DXM), the presence of anti-NMDAR antibodies in the CSF gradually decreased. The control brain MRI was negative. The patient awoke after 5 months. Neurological examination showed dysexecutive syndrome and slight tetraparesis. Her speech was limited due to long-term tracheal intubation, so she underwent an intensive logopedic intervention with orofacial stimulation therapy and swallowing rehabilitation exercises. After intensive physical therapy, she was able to walk with walking assistance devices (namely a rollator). At the time of discharge, she had achieved an improvement in her mRS score, with a score of 3 being recorded.

## 3. Discussion

Anti-NMDARE is a rare autoimmune disease associated with ovarian teratoma. It is well known that autoantibodies against the NR1 subunit play a role in the pathomechanism of NMDAR encephalitis and in the pathophysiology of schizophrenia and other psychiatric disorders [2,7]. This severe neurological disease is defined by a clinical manifestation of acute psychosis with a non-specific prodrome [1]. In our case, flu-like symptoms such as headache, fever, and joint pain were present at the beginning of the illness. After a few days, we observed psychiatric symptoms (agitation and confusion) in our patient and 6 days later she became gradually somnolent, exhibiting dyskinetic movements, spastic tetraplegia, and seizure activity.

### 3.1. Diagnostic Challenges

Anti-NMDARE could be misdiagnosed as schizophrenia because of the prodromal phase’s variable duration (due to it being a flu-like syndrome) following psychiatric symptoms. But there are several typical clinical features which can help clinicians to establish a diagnosis [4]. Continuous signs of positive symptoms (delusions, hallucinations, disorganized speech, etc.) and negative symptoms have to persist for at least 1 to 6 months in schizophrenia (Diagnostic and Statistical Manual of Mental Disorders: DSM-5 2013). In our case the psychotic symptoms were markedly fragmented, with there being a sudden onset of symptoms.

There are no typical EEG indicators for schizophrenia; we may find some slow waves, but an electrographic pattern with “extreme delta-brush” is typically missing [1]. The response to antipsychotic therapy may be poor in patients with anti-NMDARE compared to those with schizophrenia. In addition, a diagnosis of neuroleptic malignant syndrome can be made if an antipsychotic treatment is administered to patients with anti-NMDARE. In our case, it was not necessary to use this medication.

Findings of elevated protein concentrations in the CSF, including lymphocytic pleocytosis, are present in 80% of anti-NMDARE cases, but the most sensitive and specific marker for this disease is the detection of anti-NMDAR antibodies in serum and CSF [4].

### 3.2. Pathophysiological Insights

As we have mentioned before, both of these disorders have a common pathogenesis with IgG antibodies against the NR1 and NR2 subunits of NMDARs [4].

NMDARs are one of the non-selective ionotropic glutamate channels with high calcium permeability and are located throughout the cerebral cortex, including in the hippocampus. They perform a crucial role in the regulation of synaptic plasticity and synaptic transmission [8]. These hetero-tetrameric proteins are composed of NR1, NR2 (NR2A-D), and NR3(NR3A-B) subunits, usually observed to be di-heteromers (NR1/NR2A, NR1/NR2B) and tri-heteromers (NR1/NR2/NR3B). All of these subunits contain a large extracellular amino-terminal domain (N-terminal); three transmembrane segments forming an ion channel, which determine the calcium permeability of the channel; and an intracellular C-terminal domain [9,10]. The extracellular N-terminal domain of the NR1 subunit binds the glycine co-agonist, whereas the NR2 subunit has a binding place for the glutamate neurotransmitter. The maximum activation of NMDARs requires the presence of both neurotransmitters [9,11]. This activation opens up the channel pore, which determines the influx of Na^+^ and Ca^2+^ ions and enables many physiological processes such as learning and memory [9,11]. The dysfunction of NMDARs including both the NR1 and NR2 subunits leads to the dysregulation of synaptic communication between neurons, thus inducing many neurological diseases, including schizophrenia or schizophrenia-like syndromes [7,11]. Negative symptoms (blunted affect, alogia) commonly seen in schizophrenia were also noticed in our case.

NR1 subunits are situated all over the brain, while NR2 subunits are expressed in distinct places within the central nervous system due to their special functions. Both NR2A and NR2B subunits are placed in the mature cerebral cortex and hippocampus, usually as di-heteromers (NR1/NR2A, NR1/NR2B), but they can also be tri-heteromers (NR1/NR2A/NR2B) [10,12]. The NR2 subunits control protein–protein interactions and the properties of receptor transmissions. The expression of NR2B subunits increases gradually during the early stage of development and decreases with advancing age, while the expression of NR2A subunits increases over the entire course of development [12]. However, there are some differences in the receptors’ degradation process. The NR2A subunits work through endocytosis and degradation pathways that involve lysosomes. On the other hand, the NR2B subunits undergo recycling on the cell surface after endocytosis [12]. The NR2B subunits have a higher affinity for glutamate, thus leading to longer channel opening times than those for the NR2A subunits, which confer faster deactivation kinetics and considerable calcium-sensitive desensitization. An imbalance between the expression of these two subunits could be one of the key points in the pathogenesis of NMDAE [9]. The inadequate activation of NR2B subunits leads to excitotoxicity with neuron degradation, which can be observed upon MRI as frontotemporal atrophy due to the distribution of the NMDARs. Also, T2/FLAIR hyperintensities can be found in the hippocampus, brainstem, and cerebral cortex region [4]. FLAIR- and T2-weighted MRI showed a pontine hyperintense lesion in our patient.

The expression of NMDARs is not enough to induce anti-NMDARE. Chefdeville and colleagues reported that NR1 subunits of NMDARs were detected in their group of patients with teratomas associated with anti-NMDARE (55%) and also in the control group (64%) who did not have a clinical presentation of encephalitis. Thus, the production of anti-NMDAR antibodies is crucial in the pathogenesis of anti-NMDARE. They proved that there was a presence of immune cell infiltration near the neuroglial tissue in all cases of teratomas associated with anti-NMDARE and only in two cases from the control group. They supposed that the existence of nervous tissue (expressing subunits of NMDARs) in a teratoma can induce an immune reaction against the NMDARs in the central nervous system [2]. Using immunohistochemical staining, we confirmed the expression of the NR2B subunit of NMDARs in our case of ovarian teratoma. Thus, we believe that these findings played a key role in the pathogenesis of anti-NMDARE in our case. Based on our knowledge about the degradation process of NMDARs, in which the NR2 subunits undergo recycling on the cell surface, we hypothesize that it could lead to the prolonged stimulation of the immune system via excessive antibody production, including the intrathecal synthesis of antibodies.

### 3.3. Treatment Considerations

Thus, the response to standard first- and second-line immunotherapy can be insufficient with disease progression.

The efficacy of these treatments can be limited in cases presenting excessive intrathecal antibody production with immune cell infiltration near to neuroglial tissue [6]. We should have realized that many medications cannot cross the blood–brain barrier (BBB) or have very poor penetration through it to the CNS, where peripherally activated B cells undergo transformation into long-lived plasma cells with a half-life of more than 6 months, leading to prolonged therapy durations [6]. It is proposed that the intrathecal administration of methotrexate could be beneficial for treating unresponsive forms of anti-NMDARE [6,13]. Our patient had a very poor response to standard therapy, including tumor removal, showing a GCS score of 3 and an mRS of 5. We believed that, in our case, an intrathecal application of MTX would be beneficial. MTX is the chemotherapeutic agent indicated for many neoplastic diseases, such as choriocarcinoma, lymphoma, and leukemia [5,13]. The use of intrathecal application can lead to some side effects such as aseptic meningitis or leukoencephalopathy and transverse myelopathy. These side effects can be reduced when intrathecal therapy is administered in combination with DXM. DXM decreases the expression level of IL-6, an inflammatory cytokine in the CSF, which may cause nonspecific inflammatory reactions (fever, headache, lower back pain, meningism) after MTX administration [14]. No side effects were presented in our case.

The main limitation of our investigation is that it is a single case report. Further prospective randomized studies are required to identify whether our molecular findings can lead to serious immune responses in patients who do not respond to standard immunotherapy.

## 4. Conclusions

In our case, the first- and second-line immunotherapies applied following tumor removal were insufficient. Paraneoplastic anti-NMDAR encephalitis with NR2B subunit coexpression in the neural cells of ovarian teratoma may suggest a different inflammation process at work and could be a key factor in the pathomechanism and treatment of refractory anti-NMDAR encephalitis. We postulate that using additional immunohistochemical staining in clinical practice may be helpful to detect the expression of NR2B subunits in the neural cells of ovarian teratomas.

We believe that anti-NMDARE with an intrathecal synthesis of NMDAR antibodies and immunohistochemical finding of the NR2B subunit of NMDARs requires early and intense therapy rather than an escalation from first- to second-line immunotherapy. Early aggressive therapy with an intrathecal application of MTX should help to prevent poor long-term patient outcomes. This treatment strategy is based on MTX’s good penetration of the BBB.

However, more controlled trials are necessary to confirm our hypothesis and suggestion. Our findings should be considered with caution due to this being a single case report.

## Figures and Tables

**Table 1:** Therapeutic strategies based on observational studies and clinical experience.

Treatment	Dose/Unit/Frequency	Route of Administration	Duration
**First-line immunotherapy**			
Methylprednisolon	1 g daily	Intravenous	3–5 days
Immunoglobulin	2 g/kg over 5 days (400 mg/kg/Day)	Intravenous	5 days
Plasma exchange	1 session every other day	Intravenous	5–7 cycles
**Second-line immunotherapy**			
Rituximab	375 mg/m^2^/weekly	Intravenous infusion	4 weeks
Cyclophosphamid	750 mg/m^2^/monthly	Intravenous infusion	3–6 months
Tocilizumab	Initially—4 mg/kg Increase to 8 mg/kg (depending on clinical response)	Intravenous infusion	Monthly depending on clinical response
Low-dose interleukin-2	1.5 million IU/day 3 week interval (4 injections)	Subcutaneous	

*Source:* Reprinted/adapted with permission from Ref. [6]. 2018, Sage Publication.

**Table 2:** Basic cytobiochemical examination of patient’s cerebrospinal fluid.

Basic Cytobiochemical Examination of Cerebrospinal Fluid	Values
CSF Glucose	2.49 mmol/L
CSF Proteins	1.43 g/L
CSF Albumin	0.808 g/L
CSF CRP	0.050 mg/L
CSF Lactate	2.4 mmol/L
CSF Chlorides	120 mmol/L
CSF Mononuclear cells	723/µL
CSF Polynuclear	2/µL

**Table 3:** Laboratory screening of neurotropic viruses.

Etiological Agent	Diagnostic Method	Biological Material	Result
*E. coli*	PCR	Serum/CSF	Negative
*Haemophilus influenzae*	PCR	Serum/CSF	Negative
*Listeria monocytogenes*	PCR	Serum/CSF	Negative
*Neisseria meningitidis*	PCR	Serum/CSF	Negative
*Streptococcus agalactiae*	PCR	Serum/CSF	Negative
*Streptococcus pneumoniae*	PCR	Serum/CSF	Negative

**Table 4:** Timeline of the patient’s CSF findings and treatments.

	Day 1	Day 7	Day 10	Day 16	Day 22	Day 33	Day 39	Day 47	Day 53		Day 56	Day 65	Day 86	Day 101	Day 106	Day 110	Day 116	Day 132	Day 151	Day 174
CSF Proteins (g/L)	1.43	ND	0.55	ND	0.42	ND	0.25	ND	ND	CSF Proteins (g/L)	ND	0.33	0.37	ND	ND	ND	ND	1.06	ND	ND
CSF Albumin (g/L)	0.808	ND	0.212	ND	0.118	ND	0.105	ND	ND	CSF Albumin (g/L)	ND	0.1	0.115	ND	ND	ND	ND	0.594	ND	ND
CSF Mononuclear cells/µL	723	ND	533	ND	76	ND	14	ND	ND	CSF Mononuclear cells/µL	ND	7	28	ND	ND	ND	ND	0	ND	ND
CSF Polynuclear/µL	2	ND	4	ND	0	ND	1	ND	ND	CSF Polynuclear/µL	ND	0	127	ND	ND	ND	ND	1	ND	ND
CSF anti-NMDAR	ND	ND	+	ND	+	ND	+	++	ND	CSF anti-NMDAR	ND	+	++	++	ND	++	+	+	ND	ND
Serum anti-NMDAR	ND	ND	++	ND	++	ND	++	+	ND	S-anti-NMDAR	ND	-	++	++	ND	+	++	+	+	+
Acyklovir (g) (1 g/Day) Over 5 days	5	ND	ND	ND	ND	ND	ND	ND	ND	Acyklovir (g)	ND	ND	ND	ND	ND	ND	ND	ND	ND	ND
Methylprednisolon (g) (1 g/Day) Over 5 days	ND	5	ND	ND	ND	ND	ND	ND	ND	Methylprednisolon (g)	ND	ND	ND	ND	ND	ND	ND	ND	ND	ND
Immunoglobulin(g) (0.4 g/kg/Day) Over 5 days	ND	ND	200	ND	ND	ND	ND	ND	ND	Immunoglobulin (g) (2 g/kg/Day) Over 3 days	540	ND	ND	ND	ND	ND	ND	ND	ND	ND
Plasma exchange (mL)	ND	ND	ND	ND	1400	ND	ND	ND	ND	Plasma exchange (mL)	ND	ND	ND	ND	ND	ND	ND	ND	ND	ND
Rituximab (mg)	ND	ND	ND	300	ND	300	ND	ND	ND	Rituximab (mg)	ND	ND	ND	ND	ND	ND	ND	ND	ND	ND
Cyclophosphamid (mg)	ND	ND	ND	ND	ND	ND	ND	ND	ND	Cyclophosphamid (mg)	ND	ND	ND	ND	1600	ND	ND	ND	ND	ND
Methotrexate (mg) (Intrathecal)	ND	ND	ND	ND	ND	ND	ND	ND	ND	Methotrexate (mg) (Intrathecal)	ND	ND	ND	15	ND	15	10	10	ND	ND
Dexamethason (mg) (Intrathecal)	ND	ND	ND	ND	ND	ND	ND	ND	ND	Dexamethason (mg) (Intrathecal)	ND	ND	ND	20	ND	20	20	20	ND	ND
LSK ovary cystectomy	ND	ND	ND	ND	ND	ND	ND	ND	SI	LSK ovary cystectomy	ND	ND	ND	ND	ND	ND	ND	ND	ND	ND

(CSF—cerebrospinal fluid; LSK—laparoscopic; SI—surgical intervention; ND—not done). A semi-quantitative method was applied to detect the presence of anti-NMDAR antibodies in the serum and CSF. A plus means the result is positive and anti-NMDAR antibodies were detected, a minus means the result is negative.

## Data Availability

The original contributions presented in this study are included in the article. Further inquiries can be directed to the corresponding author(s).
